# Effect of Fiber Wrapping on Bending Behavior of Reinforced Concrete Filled Pultruded GFRP Composite Hybrid Beams

**DOI:** 10.3390/polym14183740

**Published:** 2022-09-07

**Authors:** Lokman Gemi, Emrah Madenci, Yasin Onuralp Özkılıç, Şakir Yazman, Alexander Safonov

**Affiliations:** 1 Meram Vocational School, Necmettin Erbakan University, Konya 42000, Turkey; 2 Department of Civil Engineering, Necmettin Erbakan University, Konya 42140, Turkey; 3 Ilgın Vocational School, Selçuk University, Konya 42615, Turkey; 4 Center for Materials Technologies, Skolkovo Institute of Science and Technology, 121205 Moscow, Russia

**Keywords:** composite concrete, composite materials, EBR method, fiber-reinforced materials, flexural strengthening, mechanical property, pultruded GFRP, FRP wrapping

## Abstract

The application of pultruded fiber reinforced polymer (FRP) composites in civil engineering is increasing as a high-performance structural element or reinforcing material for rehabilitation purposes. The advantageous aspects of the pultrusion production technique and the weaknesses arising from the 0° fiber orientation in the drawing direction should be considered. In this direction, it is thought that the structural performance of the profiles produced by the pultrusion technique can be increased with 90° windings by using different fiber types. This paper presents experimental studies on the effect of FRP composite wrapping on the flexure performance of reinforced concrete (RC) filled pultruded glass-FRP (GFRP) profile hybrid beams with damage analysis. The hybrid beams are wrapped fully and partially with Glass fiber reinforced polymer (GFRP) and carbon fiber reinforced polymer (CFRP) composites. Hybrid beam specimens with 0° to 90° fiber orientations were tested under three- and four-point bending loads. Based on the experimental load–displacement relationship results, initial stiffness, ductility, and energy dissipation capacity were compared. The experimental findings revealed that the maximum load-carrying capacities of beams produced with pultrude profiles increased by 24% with glass wrapping and 64.4% with carbon wrapping due to the change in the damages. A detailed damage analysis is provided. Similarly, significant increases were observed in structural performance ratios such as initial stiffness and ductility ratio.

## 1. Introduction

Fiber reinforced polymer (FRP) composites are one of the most important engineering elements which are extensively utilized for strengthening existing undamaged/damaged reinforced concrete (RC) structures or as a carrier element due to their favorable material properties in some engineering applications [1,2,3]. The FRPs feature high longitudinal tensile strength, non-corrosiveness, stiffness, strength-to-weight ratio, insect and fungal resistance, chemical attack resistance, low thermal transmissibility, and facile installation [4,5]. In practice, FRPs were used as external reinforcements to improve the flexural and shear-resistant capacity of conventional RC beams [6,7,8,9] or the compressive behavior of RC columns [10,11,12]. Various strengthening schemes can be used to increase the shear strength of RC beams such as full wrapping, U wrapping, and side bonded [13,14]. Besides the advantages of these applications, there are also various disadvantages. In detail, we can say the following: the full wrapping scheme in which the laminates are wrapped around the entire cross-section is the most effective method of strengthening against shear with FRP, but this scheme is impractical in terms of constructability due to the presence of monolithic slabs or other support elements. While applying FRP laminates in the form of a U-wrapping scheme is a common and effective method in shear reinforcement applications of most RC beams, FRP debonding is the main failure mode that limits the FRP shear contribution in many cases. The side bonded scheme, which involves bonding laminates only to the beam sides, does not significantly contribute to the beam loading capacity. Furthermore, the long-term performance of FRP- strengthened RC structures is also highly dependent on their interfacial bond quality as the FRP [15,16,17,18]. Literature studies have shown that the FRP and RC interfacial bond condition plays a critical role in the reinforcement effectiveness of FRP-RC beams [19,20,21,22,23,24,25,26,27,28,29].

**Figure 1:**
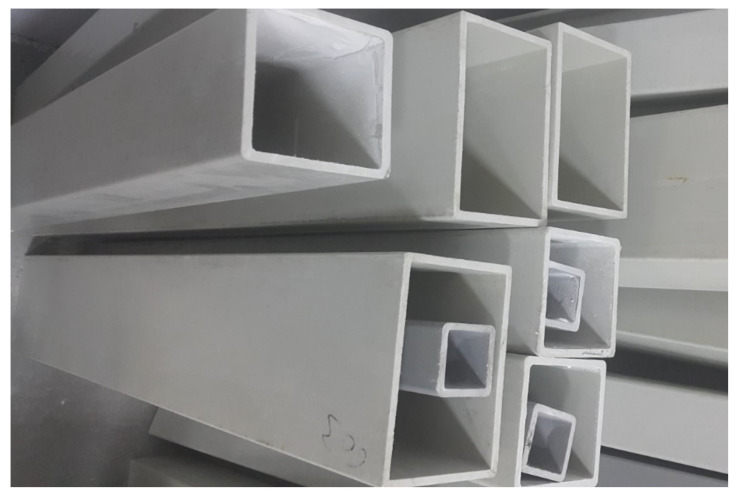
Conventional box pultruded profiles.

Recently, FRP-RC composite structural systems using concrete filled pultruded FRP profiles have become important for the construction of new high-performance structural members [30]. Pultruded profiles for commercial and structural applications are produced in conventional profile shapes similar in geometry to those of metallic materials such as the box, I-, wide flange, tube, and channel profiles (Figure 1). Pultruded profiles are made of pultruded materials which consist of FRPs (typically, glass fiber (GFRP), carbon fiber (CFRP) aramid or metallic fibers) and thermosetting resins (typically, polyester, vynilester, and epoxy polymers) [31,32]. The performance of FRP profiles depends on the fiber type. Li and Guo [33] presented a carbon/glass fiber reinforced polymer hybrid rod (HFRP) that was developed as a bridge cable to realize maximum applications [34], which combines the advantages of GFRPs with lower costs [35] and CFRPs with superior mechanical properties (stiffness and strength) [36]. It is expected that HFRPs will have excellent fatigue and corrosion resistance compared to single GFRPs. Rutkowska et al. [37] presented that the degradation of tensile properties for FRP rods was related to the cross-linking degree of resin molecular structure, and the cross-linking degree was susceptible to resin degradation. The carbon/glass hybrid rod with a diameter of 22 mm was produced through pultrusion technology for structural application by Li and Yin [38]. Hybrid FRP rods were exposed in an underground oil well with conditions including elevated temperatures, hydraulic pressure, and cyclic load coupling. Al-Mayah et al. [39] put forward an innovative pre-stressing anchor and the mechanical anchor can grip and pre-stress the CFRP plate to its full tensile capacity without any premature failure. The volume fraction of the FRP in a pultruded profile is typically between 30% and 50% [40]. In literature, it has been reported that the use of pultruded FRP profiles contributed to enhanced reinforcement effectiveness when used for strengthening of RC structures [41,42,43,44,45,46,47,48,49]. The limitations of fibers in composite mechanical properties can be improved by hybridizing different conventional materials. For example, if superior mechanical properties are to be obtained, two or more different composite and conventional materials can be hybridized. Thus, a deficient feature of one material can be complemented by a superior feature of another in the hybrid material.

The pultrusion technique is a continuous and highly cost-effective manufacturing technology for producing constant-cross section FRP structural profiles [50,51,52,53,54]. The pultrusion technique used in the production of FRP profiles is preferred due to its many advantages [55,56,57,58,59,60,61]. It is a process using oriented fibers where while most of the fibers are placed lengthwise to obtain optimum tensile strength, some fibers can be arranged in different directions to provide the desired product properties [62]. In the case of a design with FRP pultruded profiles, design-based coding based on high-level consensus developed by regulations is not yet available. The large variety of mechanical properties of various FRP materials also results in some usage limitations during the design [63]. For example, generally, GFRP materials have lower tensile modulus and strength as well as much lower material cost than those of CFRP, but the GFRPs also possess higher deformability and better impact-resistant properties [64,65]. GFRP has good energy absorption capacity and is recommended for use in earthquake-strengthening projects.

Many authors have reported or tested the mechanical properties of the components in order to analyze the mechanical behavior of pultruded FRP profiles [30,66,67]. Considering the orthotropic nature, fiber orientation has been found to have a significant influence on the material properties of laminated or pultruded FRPs [68,69,70,71,72,73]. In practice, generally, for materials with small off-axis angles (e.g., 0°), the majority of the load is taken along the fiber orientations [74,75]. At high off-axis angles (higher than 30°), the fiber has little effect and the FRP composite presents similar behavior to that of just the matrix. Due to the higher strength of the fiber, considerably higher strength or stiffness of FRP composite can be achieved in small off-axis fiber orientations compared with high off-axis loading directions [76]. In some relevant works, the differences between pultruded FRP strengths at 0° and 90° have been found. Madenci et al. [77,78] performed experimental, theoretical, and numerical analyzes to determine the mechanical properties of the pultruded GFRP composite beams in different cross sections and different fiber directions. Considering the fiber direction of 0° in the specimen which has a 0° fiber orientation, it was seen that the stress loads occurring at the bottom of the specimen came parallel to the fiber direction, and therefore, the fibers bear the entire load. In the specimen which has a 90° fiber orientation, it was observed that the 90° fiber layer had no effect on load bearing and resulted in dense debonding damage and the formation of dense matrix cracks between the fibers.

Furthermore, due to the production method of pultruded profiles, multi-directional matte felt layers converge at the corner points of the profile and discontinuities occur in the mat layer at the joints. This situation reverses the commonly known situation in many fiber orientation situations. The mat layer that surrounds the roving layers and increases the overall strength of the beam causes the corner points of the pultruded FRP profile to remain weak, but it prepares the ground for the first splitting damages. Roving layers with 0° in the pultruded FRP profile increase the strength in the profile direction, but also cause the splitting damage to progress in the profile direction. Therefore, in this study, FRP wrap applied at 90°, both delays the progression of the splitting damage and strengthens the profile section by removing the discontinuity that occurs in the mat layer with its full wrapping effect. On the other hand, despite the capability of the FRP to enhance the resistance capacities, there is often a concern that premature debonding may limit the effectiveness of the strengthening scheme.

Although the importance and suitability of RC-filled FRP profiles have been proven by many researchers, there have been limited studies investigating the use of this hybrid system for flexural elements. Parameters such as the mechanical properties of the fiber, the orientation angle of the fiber, and the loading state are the most important factors affecting the operation of RC-filled FRP profiles. Studies taking these effects into account will guide designers. This study aims to investigate the effect of FRP orientation and wrapping on the bending behavior of concrete-filled pultruded GFRP square profile beams. For this purpose, seven different combinations of RC-filled pultruded GFRP profile hybrid beams with different properties were prepared. GFRP/CFRP fabric full/partial wrapping applications in 90° FRP orientation were applied to GFRP profiles with 0° FRP orientation. Three/four-point static bending tests were conducted to evaluate the strength, stiffness, and failure mechanisms of the concrete-filled pultruded GFRP profile hybrid beams. In the experimental section, initial stiffness, ductility, and energy dissipation capacity were compared based on the load–displacement relationship. The study detailed damage analyses on which crucial failure and delamination progression on pultruded GFRP and RC beam were performed in the 4th part.

## 2. Experimental Section

In this study, the effects of CFRP and GFRP wrappings on the RC-filled pultruded GFRP box profiles were investigated. Pursuant to this goal, a total of 7 different specimens having different lengths and materials for composite wrapping were tested. The following sections provide the details of test specimens and test setup.

### 2.1. Properties of Pultruded GFRP Profile

Square pultruded GFRP box sections (100 mm × 100 mm × 6 mm thickness) produced by Pul-Tech FRP company, Turkey were used in this study. The tubes were produced using pultrusion process with vinyl ester resin and E-glass fiber reinforcement. Burnout test conducted as per ISO 1172 standard revealed that the density and the volume fraction of fiber and matrix are obtained as 54% and 46%, respectively. Table 1 shows the mechanical properties of the pultruded GFRP profiles. The elastic modulus and shear modulus of the square pultruded GFRP sections were determined previously by Madenci et al. [77,78,79] and are listed in Table 1. On the other hand, coupon tests were conducted to determine the compressive and tensile strength properties of the sections by Özkılıç et al. [64].

### 2.2. Properties of GFRP/CFRP Fabric

**Figure 2:**
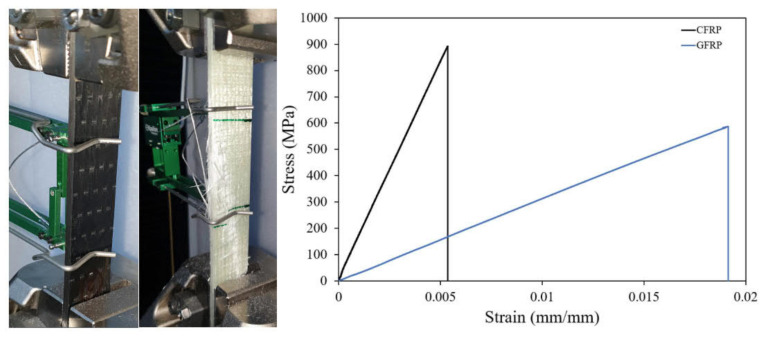
Test setup and experimental results of the coupon test.

Tensile specimens were produced to determine the mechanical properties of unidirectional carbon and glass fabrics wrapped on pultrude profiles used in the study. In the tensile test, the specimens were tested according to ASTM D3039. The size of the coupons was 250 mm × 25 mm × 4 mm. Tensile tests were carried out with a Shimadzu tensile testing machine with a capacity of 100 kN at a test speed of 2 mm/min. Test results are given in Figure 2 as stress-strain graph (CFRP-900 MPa, GFRP-600 MPa).

### 2.3. Properties of RC

The 28-day average cylinder concrete compressive strength of the concrete was calculated as 30 MPa. The maximum aggregate grain diameter of 11.2 mm (D < 12 mm) was used in order to facilitate the placement of the concrete. The longitudinal reinforcements of 3ϕ8 (ρ = 0.0195) were placed in the tension zone of all specimens (SP1–SP7). On the other hand, 2ϕ8 (ρ′ = 0.0130) longitudinal reinforcements were placed in the compressive zone for only specimens having a length of 1000 mm (SP1–SP4). Moreover, the stirrups of Ø8/200 were included for the specimens having a length of 1000 mm (SP1–SP4). The minimum and balanced reinforcement ratio in the tensile zone of a beam with C30 and B420c reinforcement class according to TS500 are ρ_min_ = 0.0028 and ρ_b_ = 0.0237, respectively. The specimens were designed to be under-balanced (ρ_min_ = 0.0028 < ρ = 0.0195 < ρ_b_ = 0.0237).

### 2.4. FRP Wrapping Application

**Figure 3:**
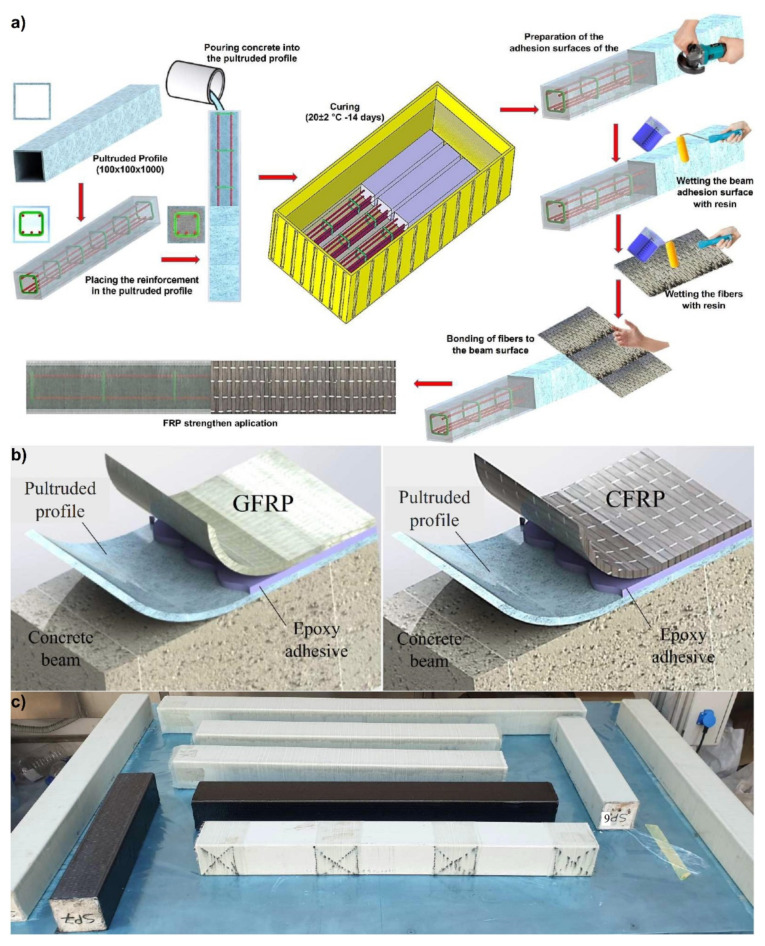
Hybrid beams production stages (**a**) schematic representation of the production process (**b**) application method of uniaxial GFRP and CFRP fabrics (**c**) The appearance of FRP applied samples during curing.

In this study, uniaxial single layer 245 g/m^2^ glass (GFRP) and 400 g/m^2^ carbon (CFRP) fiber fabric used in SP2-3-4-6-7, 90° configuration FRP wrap composites were used. F-1564 resin and F-3486-3487 hardener are preferred in GFRP wrapping applications. A mixture ratio of 100/34 resin and hardener was used upon the manufacturer’s recommendation. The fibrous fabrics to be used to strengthen the beams were cut according to the beam lengths and weighed. The determined 34% hardener was added to the resin and mixed with a mechanical mixer for 10 min. The cut fibers were soaked with the prepared resin mixture on a vacuum infusion bench and made to the consistency to be wrapped in the beams. In the resin impregnation process, sponge roller and plastic spatula are used in order not to damage the fiber fabric. Beam surfaces produced in three different reinforcement designs in pultruded GFRP profile were placed on a wet cloth cleaned with acetone and wrapped. In the winding process, the ends of the glass fabric were applied by overlapping them in such a way that the ends of the beam remained on the upper surface of the beam during the experiment. Beams wrapped in glass fabrics were allowed to cure for 10 h at 80 °C in a vacuum infusion (Figure 3c).

### 2.5. Test Specimens

**Figure 4:**
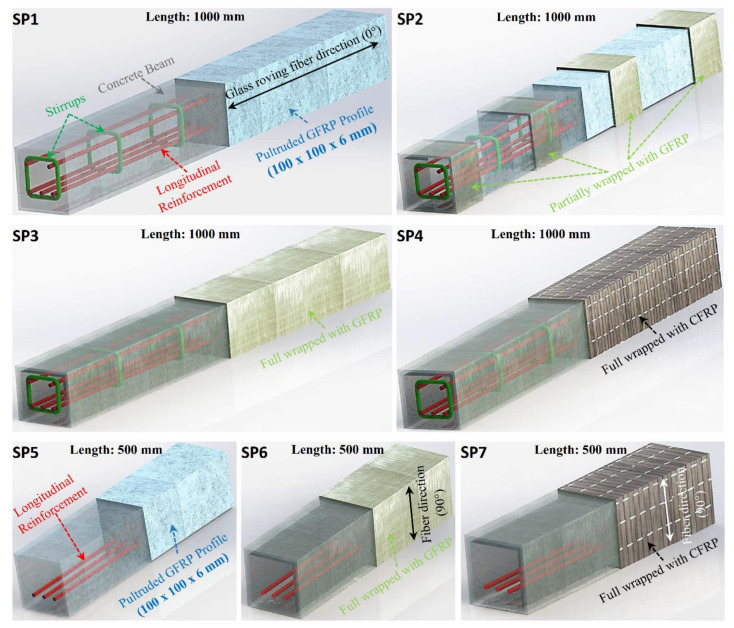
Test specimens and details of specimens.

The pultruded GFRP profiles were filled with reinforced concrete. Three longitudinal steel bars in tension zone and two longitudinal steel bars in compression zone were used for the specimens having a length of 1000 mm whereas only three longitudinal steel bars in tension zone were utilized for the specimens having a length of 500 mm. Specimens SP1–SP4 have a length of 1000 mm. Specimen SP1 represents the reference specimen for specimens SP1–SP4, which contains only pultruded GFRP and reinforced concrete. Specimen SP2 was wrapped partially with GFRP sheet under loading points and at supports. Specimen SP3 was fully wrapped with GFRP sheet whereas specimen SP4 was fully wrapped with CFRP sheet. On the other hand, Specimens SP5–SP7 have a length of 500 mm. Specimen SP5 represents the reference specimen for specimens SP5–SP7, which contains only pultruded GFRP and reinforced concrete. SP6 was fully wrapped with GFRP sheet whereas SP7 was fully wrapped with CFRP. The details of the test specimens are illustrated in Figure 4 and given in Table 2.

### 2.6. Test Setup

**Figure 5:**
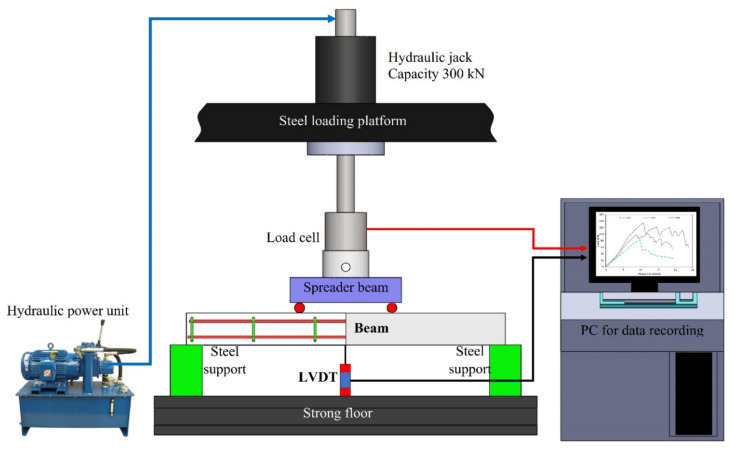
Test Setup and Instruments.

An experimental program was conducted at Necmettin Erbakan University. A total of seven specimens were tested. The experimental program was conducted using two different bending tests: three-point bending test and four-point bending test. The test specimen with length of 500 mm was tested under three-point bending while the test specimens with length of 1000 mm were tested under four-point bending. In order to measure vertical displacement, a linear variable differential transformer (LVDT) was placed in the middle of the beams. Loading was measured through a load cell which is mounted under a hydraulic cylinder. The test setup is depicted in Figure 5.

## 3. Test Results

**Figure 6:**
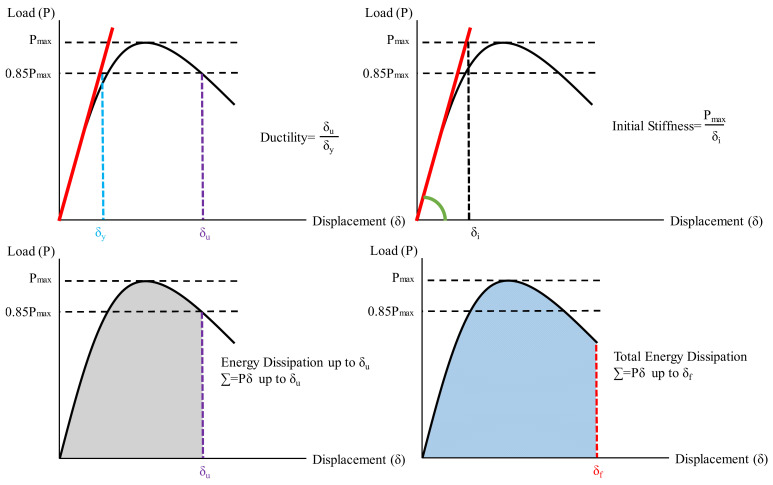
Calculation of the ductility, initial stiffness and energy dissipation.

The experimental results are summarized in Table 3 in terms of maximum load, ductility, and energy dissipation capacity. The ductility ratio was calculated as the ratio of the displacement value corresponding to 85% of the maximum loading capacity reached (δ_u_) by the beam to the displacement value at the yielding (δ_y_) [80,81]. The energy dissipation capacity is calculated as the area under the load–displacement curve. Two different energy dissipation capacities were calculated. The first one is calculated up to δ_u_ and the latter is calculated for the entire curve. Figure 6 depicts the calculation of these parameters.

**Figure 7:**
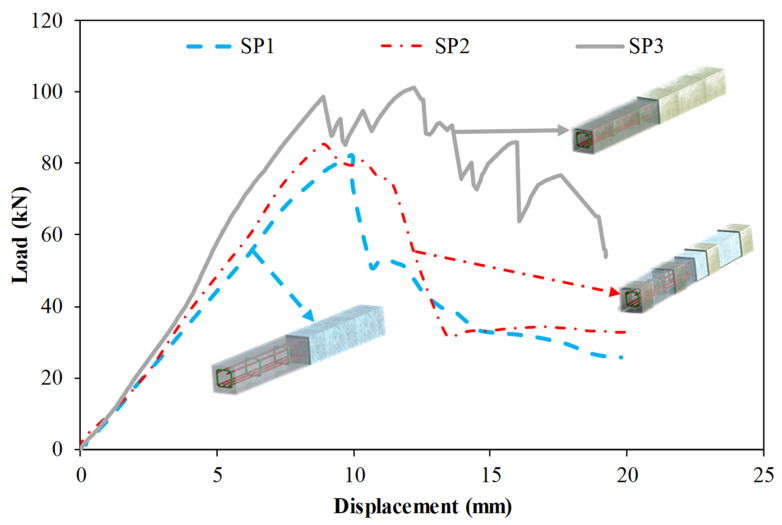
Comparison of Specimens SP1, SP2 and SP3.

Figure 7 demonstrates the results of Specimens SP1, SP2, and SP3. Specimen SP1 is the reference beam while Specimens SP2 and SP3 are the beams strengthened with GFRP composite. GFRP composite was fully wrapped to the entire specimen SP3 whereas GFRP composite was partially wrapped under loading points and at supports of Specimen SP2. Partially wrapping slightly improved the behavior of the pultruded profile infilled with reinforced concrete. Initial stiffness was increased by 16% compared to the reference specimen. A very slight difference of 4% in load-carrying capacity was observed. The difference is more pronounced in ductility and energy dissipation capacity. Ductility was increased by 15% compared to the reference specimen. Energy dissipation capacity up to δ_u_ and total energy dissipation capacity were, respectively, increased by 35% and 45%. On the other hand, full wrapping increased initial stiffness by 34% compared to the reference specimen. Moreover, for this case, load-carrying capacity significantly increased (24%). A 34% increase in ductility was observed. Energy dissipation capacity up to δ_u_ and total energy dissipation capacity were, respectively, increased by 125% and 61%.

**Figure 8:**
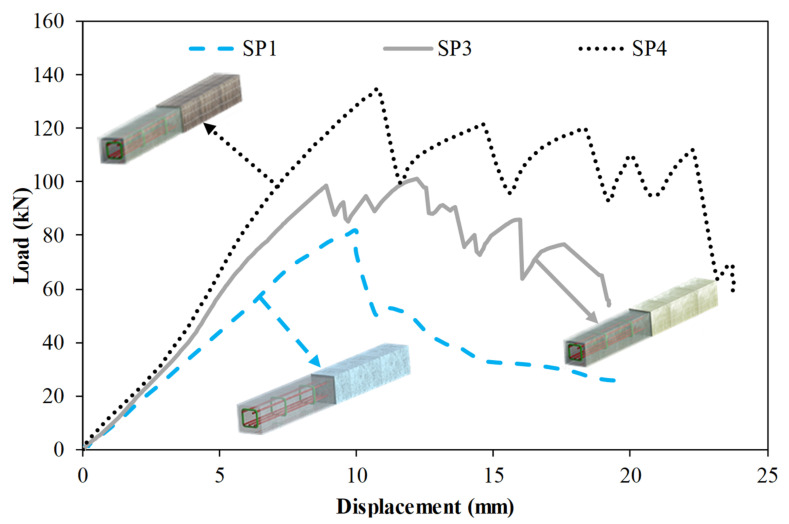
Comparison of specimens SP1, SP3 and SP4.

Figure 8 compares the specimens SP1, SP3, and SP4. Specimens SP3 and SP4 were designed to investigate the effects of wrapping composite material. Specimen SP3 was wrapped with GFRP composite while Specimen SP3 was wrapped with CFRP composite. These specimens were fully wrapped and the length of these specimens is 1000 mm. Specimen SP4 increased initial stiffness by 48% compared to the reference specimen. A 64% increase in the load-carrying capacity and ductility were observed. Energy dissipation capacity up to δ_u_ and total energy dissipation capacity were, respectively, increased by 261% and 165%. When specimen SP3 is compared to specimen SP4, it is clearly seen that the specimen with CFRP wrapping exhibited better performance than the specimen with GFRP wrapping. Specimen SP4 exhibited 11%, 33%, and 6% higher initial stiffness, load-carrying capacity, and ductility than those of Specimen SP3.

**Figure 9:**
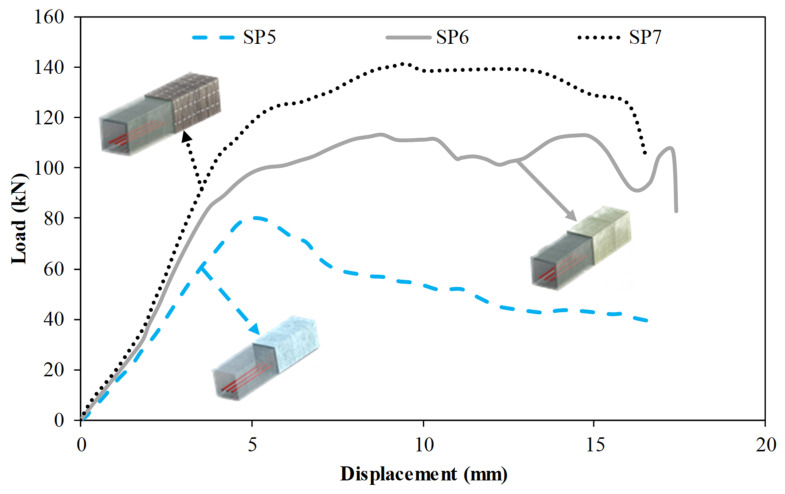
Comparison of specimens SP5, SP6 and SP7.

Figure 9 compares the specimens SP5, SP6, and SP7. Specimens SP3 and SP4 were designed to investigate the effects of wrapping composite material; however, the length of the specimens was reduced to 500 mm. Moreover, three-point loading was applied to these specimens. Specimen SP5 is the reference specimen which has a pultruded profile infilled with reinforced concrete for Specimens SP5, SP6, and SP7. Specimen S6 was wrapped with GFRP composite while Specimen SP7 was wrapped with CFRP composite. These specimens were fully wrapped. The specimens with GFRP and CFRP wrapping exhibited 44% and 65% higher initial stiffness than that of the reference specimen, respectively. Load-carrying capacity was increased by 41% and 76% for the specimens with GFRP and CFRP wrapping, respectively. It is seen in Figure 9 that composite wrapping significantly enhanced the ductility of the specimens. The specimen with GFRP wrapping exhibited slightly higher ductility than that of the specimen with CFRP wrapping. Using GFRP wrapping on the pultruded profile increased energy dissipation capacity up to δ_u_ and total energy dissipation capacity by 331% and 87%, whereas these values were modified to 404% and 118% for the specimens with CFRP wrapping.

## 4. Damage Analysis

Determining the behavior of the composite structures used in the study during the experiment and determining the damage mechanisms will play an important role in the design of future similar studies. It is possible to harmonize the behavior of hybrid beams under load, consisting of concrete, pultrude GFRP, GFRP, and CFRP wrap components, which are quite different from each other, using design flexibility. In this study; both the observations during the experiment and the damage analysis made after the experiment, the material design, and the material behavior resulting from the design were examined with a broad perspective and a damage analysis was made. The damage evolutions of each hybrid beam group examined in the context of the load–displacement graphs and Table 3 were interpreted comparatively.

**Figure 10:**
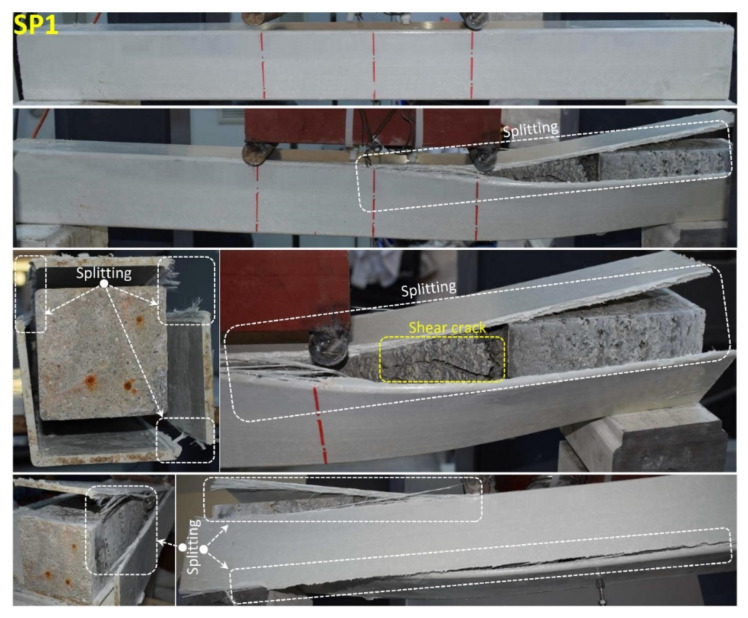
Undamaged and damaged specimen images of SP1.

When the SP1 specimen given in Figure 10 is examined together with Figure 7; it has been observed that concrete and pultrude GFRP profiles carry loads together up to 81.8 kN load level. At this load level, shear damage occurred in the concrete in the right opening of the beam with a displacement of 9.9 mm and sudden splitting damage occurred in the upper corners of the GFRP profile. The load carrying capacity decreased to 50.8 kN load level, and when the loading continued, the splitting damages continued until the compression point and continued with the load drops. Similar splitting damage at the corners was also reported by Ferdous et al. [82] who tested pultruded GFRP box profiles infilled with geopolymer concrete and by Aydın [83] who tested pultruded GFRP infilled with reinforced concrete.

**Figure 11:**
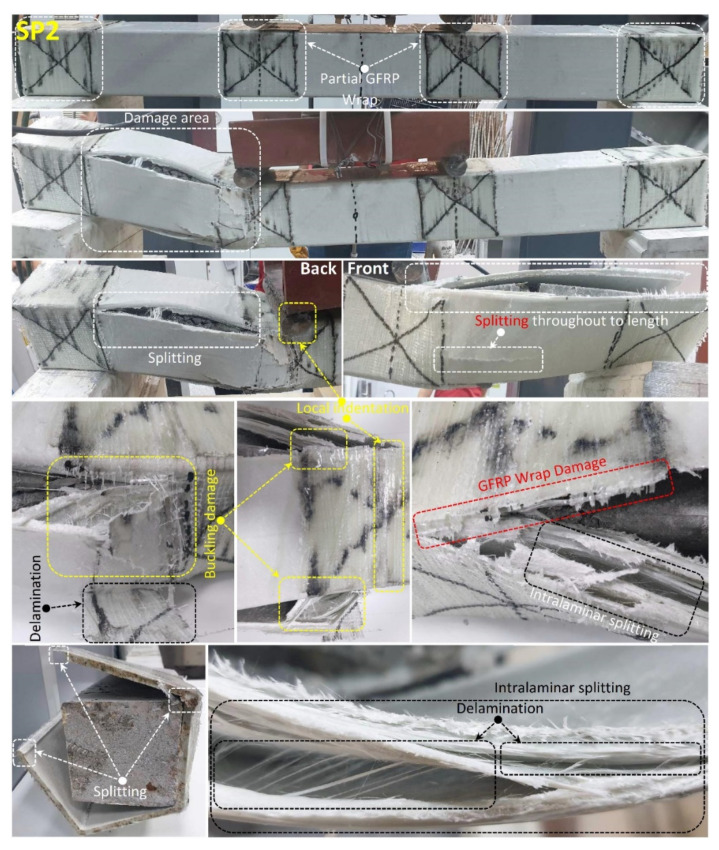
Undamaged and damaged specimen images of SP2.

In addition to the SP1 specimen, when the piece-wound SP2 specimen given in Figure 11 is examined; it was observed that shear damage in the concrete in the left span of the beam at 85.3 kN load and 9.6 mm displacement level, together with the first splitting damage occurred at the corner points of the beam between the two-piece wrapping.

After the splitting damage formation, loading was continued and it was observed that the resulting rupture damages progressed to the segmented windings with a load of 74 kN and a displacement of 12.5 mm. It has been observed that buckling occurs on the side walls of the pultruded GFRP profile with the effect of the GFRP winding, and with the effect of these buckling, intra-layer delaminations occur especially in the buckling region concentrated at the compression point. Splitting damages at the corner points of the profile forced the GFRP winding areas with the increase in displacement and with a displacement of 13.4 mm. It has been observed that the segmented GFRP winding at the compression point and the end of the beam gradually cuts through.

**Figure 12:**
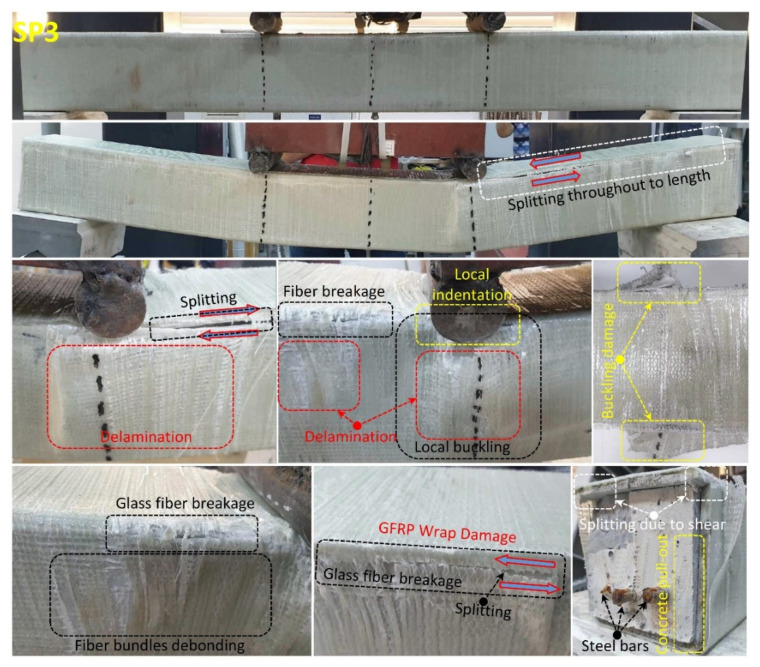
Undamaged and damaged specimen images of SP3.

**Figure 13:**
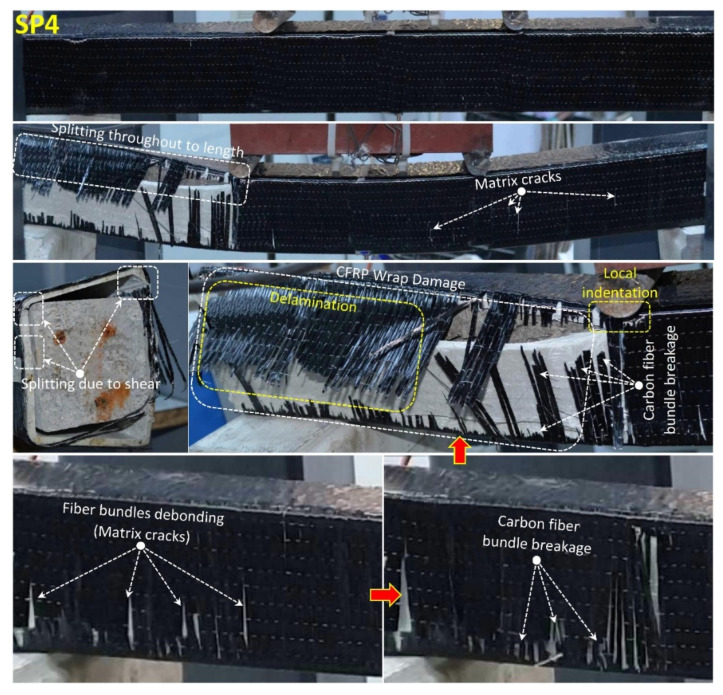
Undamaged and damaged specimen images of SP4.

When the fully wrapped SP3 specimen given in Figure 12, which is designed differently from the SP1 and SP2 specimens, is examined, it is seen that the damage behavior of the hybrid beam has completely changed. With the effect of partial and full GFRP wrapping applied to the SP1 specimen, it was understood that the stiffness and load-bearing capacity of the specimen increased gradually and the damage behavior of the concrete changed. In this specimen; it is seen that concrete and composite components work together up to a load of 98.8 kN and a displacement of 8.9 mm. It was observed that from this load level up to 101.4 kN load and 12.2 mm displacement, damages occurred in the concrete filling of the hybrid beam. The formation of damage in the pultruded profile was limited by the GFRP wrapping effect, and this prevented the shear cracks expected to occur in the concrete filling, causing the beam to exhibit ductile behavior mainly by the formation of tensile cracks. After exceeding the 101.4 kN load level, splitting damages were first observed at the upper corner points of the GFRP profile in the right span of the hybrid beam. With the increase in displacement, the splitting damages in the other corners of the beam continued to progress up to 14.5 mm displacement and 72.9 kN load level. After the splitting damages in the pultruded profile completed their development, they started to force the GFRP wrap region with the shear stresses they created at the corner points of the beam. With the effect of GFRP winding, the load-carrying capacity increased up to 86 kN. At this load level, starting from the local indentation zones formed at the compression points of the beam, the GFRP windings started to break gradually in the cleavage region formed in the right opening of the beam. Catastrophic damages that occurred in SP1 and SP2 specimens did not occur in SP3 specimens, whereas the damage occurred gradually in hybrid beam components. With the winding effect, the dispersion of the beam components is also prevented. After the test, when the cross-section of the beam is examined; pull-out damages were observed on the upper plate part of the pultruded profile and on the concrete. Splitting damage throughout the length of pultruded profile wrapped with GFRP was also observed by Gemi et al. [79] who investigated the pultruded profiles with traditional reinforced concrete. Unlike the SP3 specimen, CFRP full wrap was applied instead of GFRP and an SP4 specimen was produced. The post-test damages of the SP4 specimen are given in Figure 13. As can be seen in Table 4 and Figure 8, significant increases in all mechanical properties were realized with the effect of CFRP wrapping. The CFRP winding had an effect on the damage development of the composite beam as well as its damage development differing compared to the GFRP. Unlike the other specimens, the SP4 specimen showed a homogeneous deflection in the beam until the moment of damage. In the CFRP winding with 60.6 kN load at a 5 mm displacement level, matrix crack sounds began to be heard between fiber bundles. This situation continued until splitting damage occurred in the first pultrude GFRP at a load level of 134.5 kN starting from the compression point in the left span of the beam. CFRP winding worked with a pultrude profile up to 134.5 kN load level and 10.8 mm displacement. A gradual damage development was observed in the CFRP winding with increasing displacement after splitting damages at the corners of the pultrude GFRP profile (Figure 13).

Matrix cracks formed between fiber bundles progressed towards debonding damage. After the 19 mm displacement value, the fiber bundles started to break and this situation continued until the 64 kN load 23 mm displacement value. After this stage, a sudden severe collapse occurred in the beam, and delamination damages occurred between the pultrude GFRP profile and the CFRP winding.

**Figure 14:**
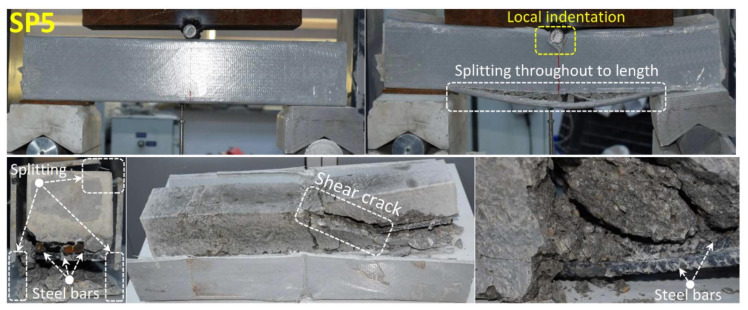
Undamaged and damaged specimen images of SP5.

**Figure 15:**
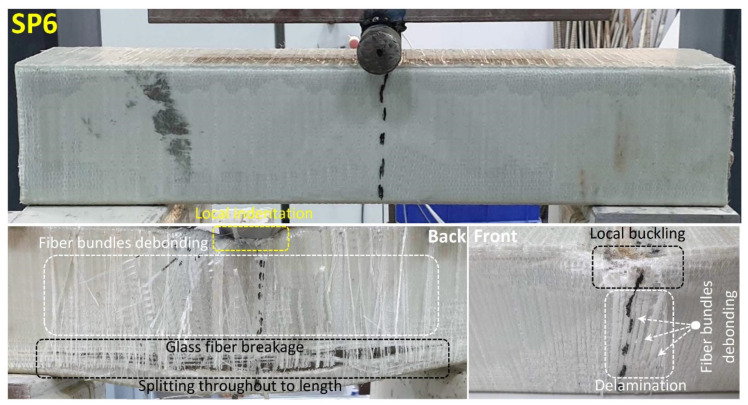
Undamaged and damaged specimen images of SP6.

**Figure 16:**
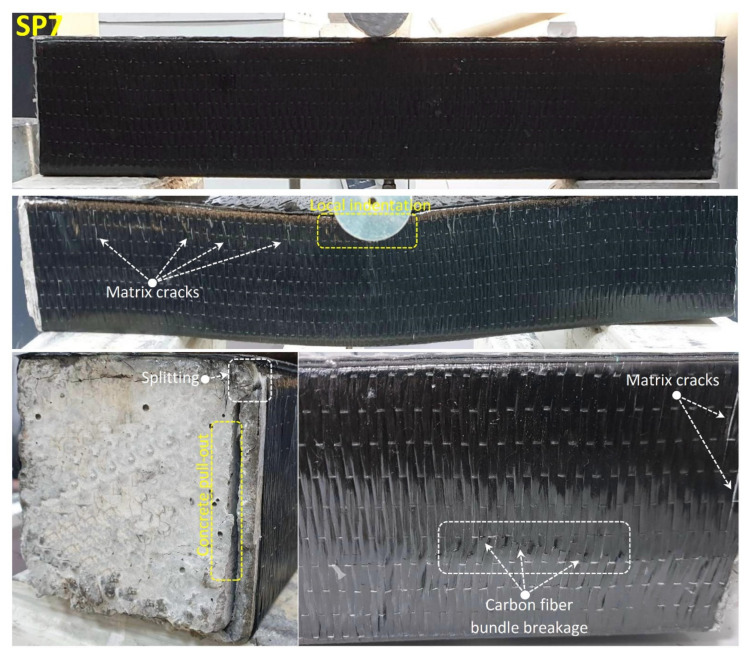
Undamaged and damaged specimen images of SP7.

GFRP and CFRP full wraps were applied under the same conditions to 0.5 m-long pultrude GFRP specimens without stirrups reinforcement and were investigated as a separate group. Three-point bending tests were carried out to compare these specimens among themselves. The damage evolutions of each hybrid beam group are examined in accordance with the load-displacement graphs in Figure 9, Table 4, and Figure 14, Figure 15 and Figure 16 were interpreted comparatively. When evaluated in general, it was observed that the mechanical properties of SP6 and SP7 specimens with glass and carbon wraps were significantly increased with the effect of the wrapping when compared to the SP5 beam. When the SP5 specimen given in Figure 14 is examined together with Figure 9, it is seen that concrete and pultrude GFRP profiles worked together up to 80 kN load and 5 mm displacement. With the shear damage in the concrete with a displacement of 5 mm, the load decreases. At this stage, local buckling occurred in the GFRP profile due to the increase in displacement and local indentation at the compression point. At the bottom of the GFRP profile with a load of 70 kN and a displacement of 6 mm, there was longitudinal splitting damage between the two supports. Similar splitting damage at the mid-section due to flexural loading was also observed by Zhang et al. [84] who infilled pultruded GFRP box with seawater and sea sand concrete.

Damage analysis photos of the SP6 specimen obtained by applying 90° GFRP wrapping on the SP5 specimen are given in Figure 15. The damage stages in the SP6 specimen were similar to the SP5 hybrid beam.

In this specimen, despite the damage to the concrete and the profile, it was observed that there was no sudden drop in load due to the GFRP wrapping effect. With the effect of local buckling at the compression point with a load of 113 kN and a displacement of 8.8 mm, delamination damage and fiber bundle debonding were observed in the GFRP wrapping. With these damages, fluctuations in the load started to occur, and this situation continued up to 14.8 mm displacement with concrete damage and splitting damage in the GFRP profile. At the 106 kN load and 17 mm displacement value, the resulting damage has occurred along with the sudden ruptures (glass fiber breakage) in the GFRP winding in the splitting damage area at the bottom of the beam.

Similarly, damage analysis photos of the SP7 specimen obtained by applying 90° CFRP wrapping on the SP5 specimen are given in Figure 16. Damages that occurred in the concrete and pultrude profile in the SP7 specimen showed damage in the order of the SP5 and SP6 specimens. However, the effect of the winding made of carbon fiber made the beam more rigid and limited damage occurrences but also delayed it. In CFRP wrap with 126 kN load and 6.4 displacement value, matrix cracks started to appear between fiber bundles, and damage to the concrete caused fluctuations in the load. Concrete pull-out damages were observed at the ends of the beam with the effect of shear damage and displacement increase in the concrete. This situation continued until the splitting damage occurred in the pultruded profile at 141.4 load and 9.5 mm displacement. It was observed that fiber bundle breakage damage started to occur in the fiber bands in certain parts of the CFRP wrap at a displacement value of 14 mm. Due to this damage, which started to spread throughout the specimen, a sudden decrease in the load started with the advancing displacement. At this stage, the experiment was terminated. Despite the damage to the GFRP wrap, the use of carbon fiber in the wrap preserved the integrity of the specimen. The observed damages are summarized in Table 4.

## 5. Conclusions

Pultruded GFRP profiles, which are widely used in many engineering fields, have recently been used as a construction material in the field of civil engineering. In recent years, the use of pultruded GFRP profiles as concrete-filled RC beams and columns has increased. Especially in beam applications, although the profiles reinforced in the tension direction significantly increase the load-bearing capacity of concrete-filled beams, they exhibit brittle fracture behavior with splitting damages in the direction of fiber reinforcement at the damage stage. It is known that utilizing a pultruded profile significantly improves the performance of the pultruded profile [79]. In this study, the load-bearing capacities of hybrid composite beams were significantly increased by applying the GFRP and CFRP winding technique perpendicular to the pultruded GFRP fiber orientation (90°). However, with high displacements, the damage development has been made progressive and the formation of sudden collapses in the beams has been prevented. Comparative comments in the study were made with reference to the SP1 specimens.

- Partial GFRP, GFRP, and CFRP wrapping effect increased the initial stiffness of beams of 1000 mm length by 15.6%, 33.7%, and 48%, respectively. In 500 mm beams, GFRP and CFRP increased the initial stiffness with a full wrapping effect of 44.2% and 65.4%, respectively. When the load carrying capacities are compared to beams of 1000 mm length; the partial GFRP, GFRP, and CFRP wrap effect increased by 4.3%, 24%, and 64.4%, respectively. These results indicate that the CFRP wrapping application is more effective than the GFRP wrapping application.
- Splitting damages were dominant especially in the corner regions of pultruded GFRP profiles while local buckling damages were dominant in the compression regions of the indentor in the wrapped samples and the cleavage damages starting from the buckling regions under the FRP wrappings progressed. Delamination, fiber bundle debonding, and fiber breakage damages in GFRP and CFRP wrappings were concentrated in the local buckling damage area. Fiber breakage damages begin with the effect of splitting due to shear damages in pultruded GFRP profiles.
- Using FRP wrapping on pultruded beams infilled with reinforced concrete significantly improved ductility, load capacity, and energy dissipation capacity. These gains can be improved by implementing a better interface with reinforced concrete [85] and the pultruded profile and by utilizing different stacking sequences of FRP wrapping [26].

## Figures and Tables

**Table 1:** Mechanical properties of pultruded GFRP.

Property	Mean Value (MPa)
Longitudinal tensile modulus of elasticity	23,000
Transverse tensile modulus of elasticity	7000
Longitudinal tensile strength	240
Transverse tensile strength	50
Longitudinal compressive strength	150
Transverse compressive strength	70
Shear strength	25

**Table 2:** Details of specimens.

Specimens	Stirrups, mm	Length, mm	FRP Strengthening
SP1	Ø8/200	1000	No
SP2	Ø8/200	1000	Partial GFRP
SP3	Ø8/200	1000	GFRP
SP4	Ø8/200	1000	CFRP
SP5	No	500	No
SP6	No	500	GFRP
SP7	No	500	CFRP

**Table 3:** Experimental results.

Test Specimens	Configuration	Initial Stiffness (kN/m)	Max Load (kN)	Ductility Ratio (δ_u_/δ_y_)	Energy Dissipation Capacity up to δ_u_ (kN·mm)	Total Energy Dissipation Capacity (kN·mm)
SP1	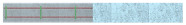	8.3	81.8	1.38	478	1049
SP2	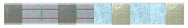	9.6	85.3	1.59	643	1173
SP3	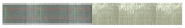	11.1	101.4	2.13	1077	1303
SP4	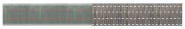	12.3	134.5	2.26	1729	2139
SP5	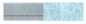	15.6	80.2	1.69	352	838
SP6	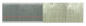	22.5	113.1	3.25	1518	1570
SP7	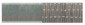	25.8	141.4	3.08	1773	1831

**Table 4:** Summary of the damages observed in the experiments.

Test Specimens	Damage Modes	Explanation
SP1 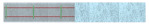	- Splitting - Shear cracks	Splitting: It is a type of matrix cracking in the direction of the fiber and parallel to the direction of the applied load. Shear cracks: Shear damage occurred in the concrete Intralaminar splitting: It is longitudinal cleavage damage that occurs in the fiber direction in pultruded GFRP. Buckling damage: It is the damage that occurs under the compression zones in hybrid beams. They are locally pultruded GFRP-based buckling damages. They are the kinkband formed inside the laminate when considered in micro dimension. Local indentation: It is the damage caused by penetrating the composite material in the force region applied by the indentor to the material in four and three point bending tests. Delamination: It is the layer separation between the pultruded CFRP reinforced in different directions and the fiber wrapping application. Fiber breakage: It is the fiber breakage caused by the cleavage damage of the applied CFRP and GFRP fiber wrappings at the corners of the Pultruted GFRPs. Debonding: It is the fiber matrix interface damage that occurs in the direction of reinforcement under load in CFRP and GFRP fiber wrappings. Matrix cracks: In CFRP and GFRP fiber wrapping applications, it is the matrix damage that occurs between the fiber bundles due to the displacement in the beam under load. Fiber bundle breakage: It is the damage of longitudinal bundle breaking of fibers after matrix cracking and debonding damages in fiber wrapping applications. Fiber bundle debonding: It is the damage caused by the progression of matrix cracks during loading in fiber wrapping applications and the separation of fiber bundles from each other. Concrete pull-out: It is formed between pultruded CFRP and concrete. It is the protrusion of the concrete at the beam ends with the effect of shear damage and displacement increase in the concrete.
SP2 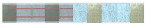	- Splitting - Intralaminar splitting - Buckling damage - Local indentation - Delamination - Fiber breakage	
SP3 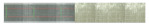	- Splitting - Intralaminar splitting - Buckling damage - Local indentation - Delamination - Fiber breakage - Debonding	
SP4 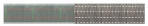	- Splitting - Matrix cracks - Fiber bundle breakage - Fiber bundle debonding - Delamination - Local indentation	
SP5 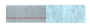	- Splitting - Shear cracks - Local indentation	
SP6 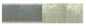	- Local buckling - Fiber bundle delamination - Delamination - Fiber breakage	
SP7 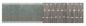	- Splitting - Matrix cracks - Fiber bundle breakage - Concrete pull-out	

## Data Availability

Not applicable.
